# Effects of SLIRP on Sperm Motility and Oxidative Stress

**DOI:** 10.1155/2020/9060356

**Published:** 2020-10-13

**Authors:** Dan Shan, Samuel Kofi Arhin, Junzhao Zhao, Haitao Xi, Fan Zhang, Chufang Zhu, Yangyang Hu

**Affiliations:** ^1^Department of Obstetrics and Gynecology, The Second Affiliated Hospital of Wenzhou Medical University, Wenzhou, Zhejiang 325027, China; ^2^Department of Physician Assistant Studies, School of Allied Health Sciences, University of Cape Coast, PMB, Cape Coast, Ghana

## Abstract

**Background:**

Deficient spermatozoon motility is one of the main causes of male infertility. However, there are still no accurate and effective treatments in a clinical setting for male asthenospermia. Exploring the genes and mechanism of asthenospermia has become one of the hot topics in reproductive medicine. Our aim is to study the effect of SLRIP on human spermatozoon motility and oxidative stress.

**Methods:**

Sperm samples were collected including a normospermia group (60 cases) and an asthenospermia group (50 cases). SLIRP protein expression in spermatozoa was examined by western blotting, and relative mRNA expression of SLIRP in spermatozoa was quantified by reverse transcription polymerase chain reaction. Levels of reactive oxygen species (ROS), adenosine triphosphate (ATP) content, and the activity of manganese superoxide dismutase (MnSOD) in spermatozoa were also measured.

**Results:**

The mRNA level and protein expression of SLIRP in the asthenospermia group were significantly reduced compared with those in the normospermia group. The ROS active oxygen level in the asthenospermia group significantly increased; however, the ATP content decreased significantly as well as the activity of MnSOD.

**Conclusion:**

SLIRP regulates human male fertility, and SLIRP and sperm progressive motility are positively correlated. The expression of SLIRP is declined, oxidative damage is increased, and energy metabolism is decreased in spermatozoa of asthenospermia patients compared to normospermia participants.

## 1. Introduction

About 10%-15% of couples of reproductive age have fertility disorders worldwide, and according to incomplete statistics, about 40% of infertility is related to male factors. Sperm motility deficiency is one of the main causes of male infertility. Mitochondrial oxidative phosphorylation (OXPHOS) is the prime source of energy produced by sperm. Insufficient energy production is due to damage of mitochondrial structure and function which probably contributed to poor sperm motility [1, 2]. Researchers have pointed out that 80% of the asthenospermia men have higher ROS levels than normal, and excessive ROS will lead to severe spermatozoa damage [3, 4].

Hatchell et al. [5] first reported the SLIRP gene in 2006, which is an RNA-binding protein on steroid receptor RNA activator (SRA), and its expression is ubiquitous in human normal tissues. Most of SLIRP is present in mitochondria, and the size of the protein that the SLIRP gene encodes is 109aa. SLIRP can affect mitochondrial mRNA transcription and energy conversion. Researchers [6] made model mice with SLIRP gene knockout to see if they could survive, but their fertility was evidently weakened and their sperm motility was deficient. In addition, the loss or weakening of SLIRP destroys part of the structure of mitochondria, affects mitochondrial energy metabolism, and increases sperm or cell oxidative stress damage.

Our research intended to explore the expression of SLIRP in human sperm and the effect of SLIRP on sperm motility and energy metabolism.

## 2. Materials and Methods

Male subjects aged 20–45 years attending the infertility clinic at the Reproductive Medicine Center of the Second Affiliated Hospital of Wenzhou Medical University were recruited for the study after obtaining informed written consent. Males with conditions that are likely to affect the sperm DNA integrity such as underlying diseases (high blood pressure, diabetes, heart disease, etc.), history of smoking/alcohol intake, history of mumps, varicocele, cancer, or history of/ongoing radiotherapy or chemotherapy were excluded from the study.

The semen samples were collected, based on the standards prescribed by the WHO, by the method of masturbation into a sterile and labeled container following an abstinence period of 2–7 days. The samples were allowed to liquefy at room temperature for 30 min before analysis. Participants were grouped into a normospermia semen group (60) and an asthenospermia semen group (50) by computer-aided sperm analysis (CASA, IVOS, Hamilton, USA). The semen analysis was performed manually based on the 2010 WHO standard protocols. The parameters—semen volume, pH, sperm motility and vitality, and sperm concentration—were analyzed. Sperm morphology was analyzed by pap stain, and DNA fragmentation analysis was performed by manual counting under the microscope after SCD assays. Detailed semen parameters were obtained (Table 1).

### 2.1. Reverse Transcription Polymerase Chain Reaction

RNA was extracted from sperm samples with an Ultrapure RNA Kit (CWBIO, China). Reverse-transcribed cDNA was then synthesized according to the HiFiScript cDNA Synthesis Kit (CWBIO, China). Regarding cDNA as a template, the detection was carried out by using the fluorescence quantitative PCR instrument (CFX Connect™, Bio-Rad, China). The SLIRP primer sequences are as follows: F:5′AACACTTTGCACAGTTCGGC3′ and R:5′GTGAACCTGGACCTTTACTCCA3′ (Sangon Biotech, China). The reagents were added according to the operation manual procedure with a total volume of 20 *μ*L. The relative expression of SLIRP in each group of sperm was then calculated by the 2^-*△△*Ct^ method with GAPDH as an internal reference.

### 2.2. Western Blotting

400 *μ*L lysis solution (Applygen, China) was added to a sperm precipitate on ice for 30 min, then centrifuged at 12000g/min for 10 min at 4°C. The supernatant was obtained for protein concentration detection according to the BCA Protein Assay Kit (CWBIO, China). The protein was denatured, loaded, and subjected to sodium dodecylbenzene sulfonate gel electrophoresis 60 V for 30 min and then 80 V for 2 h and then transferred to the membrane for 1 h. The primary antibody (1 : 1000, Bioss, China) was incubated at 4°C overnight, and then, the secondary antibody (1/2000, ZSBiO, China) was incubated at room temperature for 2 h. ECL exposure solution was added dropwise on the membrane and exposed in the gel imaging system, and finally, each antibody band was analyzed for the gray value with Quantity One software.

### 2.3. Flow Cytometry Detecting ROS Levels

Sperm ROS level was detected with a ROS Detection Kit (KeyGen Biotech, China). The sperm samples were washed with PBS and resuspended at a concentration of 10^6^ sperm/mL, and we selected 300 *μ*L for ROS level assessment. It was incubated with 10 *μ*mol/L DCFH-DA at 37°C for 20 minutes. After washing, centrifuging, and resuspending, it was then analyzed by flow cytometry (NovoCyte 2060R, ACEA BIO, China) and the mean fluorescence intensity was measured.

### 2.4. MnSOD Activity

Activity of MnSOD was measured with a human manganese superoxide dismutase kit (MEIMIAN, China) and expressed in units mg^−1^ of protein. The sperm precipitate was incubated with lysis buffer on ice for 30 min, then centrifuged at 12000g/min for 10 min at 4°C for the supernatant. The protein concentration was determined according to the BCA Protein Assay Kit (CWBIO, CHINA). 40 *μ*L of sample diluent was added to the well in a plate which was enzyme-coated; then, 10 *μ*L of the sample and 100 *μ*L of the enzyme-labeled reagent were added, and the plate was then sealed and incubated at 37°C for 60 min. Finally, a color reagent to measure the absorbance (OD value) of each well at 450 nm wavelength with the multifunctional microplate reader (Tecan, USA) was added. The activity of MnSOD was calculated according to the standard curve and protein concentration.

### 2.5. ATP Content

The ATP content of spermatozoa was determined with an ATP detection kit (Beyotime, China). ATP lysis solution was added to the spermatozoon sample on ice and then centrifuged at 12000g for 5 minutes at 4°C. The supernatant was collected for the ATP assay (*μ*mol/mg). 40 *μ*L of sample supernatant and 100 *μ*L of ATP detection working solution were added together into a 96-well plate for detection with a multifunctional microplate reader (Tecan, USA).

### 2.6. Statistical Analysis

Data were statistically analyzed with SPSS 19.0, the *t*-test was utilized to analyze the difference between the two groups, and the chi-square test was utilized to compare the rate. *P* < 0.05 was considered to be statistically significant. Quantitative data were expressed as mean ± standard error of the mean (SEM).

## 3. Results

### 3.1. Expression of SLIRP in Human Sperm and Its Relationship with Sperm Motility

Table 1 shows that there was no significant difference in participants' age, abstinence time, and semen volume between the two groups. The semen concentration was within the normal range, but 52.91 ± 25.26∗10^6^/mL in the asthenospermia group appeared to be much lower than that in the normospermia group. The proportion of progressive motility and total motility in the asthenospermia group which was 17.95% and 25.87%, respectively, showed that it was significantly worse than that in the other group. There is no obvious difference in the proportion of normal morphology sperm: 3.28% in the normospermia group and 1.99% in the asthenospermia group. The sperm DNA fragmentation rate in the asthenospermia group was 23.36%, which was evidently higher than that in the normal group.

**Figure 1:**
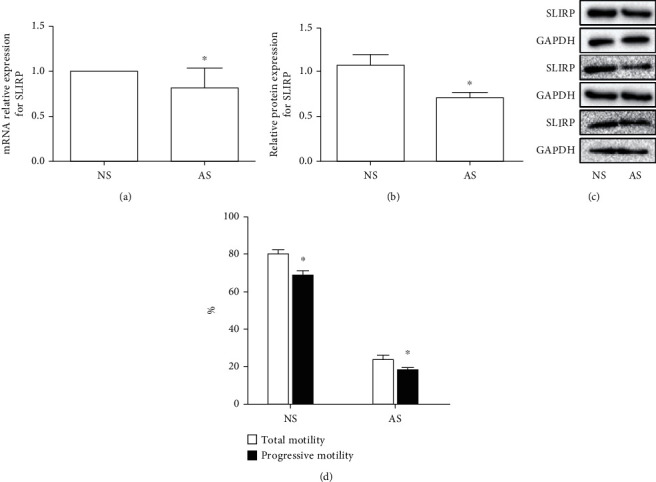
(a) mRNA relative expression for SLIRP in sperm by RT-PCR. (b, c) Relative protein expression for SLIRP in sperm by western blotting. The values were normalized to GAPDH control, and means ± SD were calculated. (d) Total motility and progressive motility of sperm. NS: normospermia group; AS: asthenospermia group. ^∗^Compared to the normospermia group (^∗^*P* < 0.05).

The researchers analyzed the mRNA expression and protein content of SLIRP in mouse spermatozoa to explore the relationship between SLIRP and sperm motility, but no research on human spermatozoon SLIRP expression was found in literature. Our study discovered that the SLIRP gene exists in human spermatozoa and its expression in asthenozoospermic sperm decreased mRNA level (Figure 1(a)) and protein contents (Figures 1(b) and 1(c)) compared with that in normal semen. Progressive motility and total motility in the asthenospermia group (17.95% and 25.87%, respectively) showed to be significantly worse than those in the normospermia group (68.52% and 80.12%, respectively) (Figure 1(d)). This shows that SLIRP is positively correlated with sperm motility (Figure 1).

### 3.2. Relationship between Spermatozoon Motility and ROS Content, ATP Content, and MnSOD Activity

**Figure 2:**
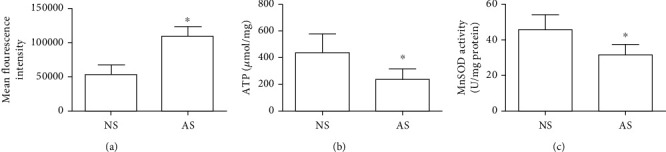
(a) ROS content was determined by flow cytometry in the expression of the mean fluorescence intensity (MFI) of sperm with the DCFH-DA probe. (b) ATP content was expressed as *μ*mol/mg protein. (c) MnSOD activity was expressed as U/mg protein. NS: normospermia group; AS: asthenospermia group. ^∗^Compared to the normospermia group (^∗^*P* < 0.05).

The ROS level was detected with the fluorescent probe DCFH-DA, and the mean fluorescence intensity (MFI) was measured with the flow cytometer. The MFI in AS was 108668 ± 25425, which was much higher than that in the NS group whose MFI was 52835 ± 16610 (*P* < 0.05) (Figure 2(a)). It is shown that the sperm motility and ROS level were negatively correlated. ATP content in AS (234.74 ± 81.21 *μ*mol/mg protein) was significantly less than that in NS (437.38 ± 142.54 *μ*mol/mg protein) (*P* < 0.05) (Figure 2(b)). Energy production in the AS group was obstructed by poor sperm motility. MnSOD activity in NS (45.31 ± 8.60 U/mg protein) appeared to be higher than that in AS (31.46 ± 5.69) (*P* < 0.05) (Figure 2(c)). The activity of MnSOD in asthenospermia sperm appeared weakened.

## 4. Discussion

SLIRP has a complex relationship with nuclear receptor target genes on the nucleus [7]. Nuclear receptors and their regulatory factors play an important role in the direct expression of primitive genes in mammalian reproduction, development, and metabolism. SLIRP is especially highly expressed in tissues with high energy requirements, so it is abundant in the testis, liver, skeletal muscle, and heart. SLIRP is also present in breast cancer, prostate cancer, and lung cancer tissues, and its expression is significantly enhanced in cancer tissues [5]. Knocking out SLIRP or LRPPRC in a model mouse results in a decrease in mRNA levels, but it does not affect tRNA and rRNA. It implied that the LRPPRC/SLIRP complex played a special role in further maturation and maintained the stability of mitochondrial mRNA after transcription [8, 9].

The testis of a mouse is rich in SLIRP. The acrosome, tail, and middle mitochondria of mature sperm are also rich in SLIRP. The average size of offspring born from homozygous SLIRP-/- male mice mating with wild-type female mice compared with those born from wild-type male mating female mice decreased about 1/3. Simultaneously, the progressive motility sperm of SLIRP-/- mice significantly declined compared to that of the control group [6]. It is observed that the middle spermatozoa and the junction between the middle and main segments of the model mice were destroyed and the mitochondrial morphology also changed when observed under an electron microscope. It can be said that SLIRP regulates male fertility, and its loss will break the sperm structure and change the mitochondrial morphology, resulting in the occurrence of asthenozoospermia.

Our study confirmed that the SLIRP gene exists in human sperm. Compared to the normospermia group, SLIRP mRNA level and protein expression in sperm in the asthenospermia group were obviously decreased; also, the sperm progressive motility significantly declined. SLIRP expression was positively correlated with sperm progressive motility (Figure 1). The sperm DNA fragmentation rate was apparently increased in the asthenozoospermia group, which was negatively related to the SLIRP gene (Table 1). We did not find any difference between asthenozoospermic patients and normal men through the analysis of apparent sperm morphology (Table 1). It turned out that asthenozoospermic patients produce more ROS (Figure 2(a)), and it was also indicated that the oxidative stress response in the asthenozoospermic patients was more intense. High levels of ROS can break DNA, cause DNA damage, and induce sperm apoptosis [10]. Excessive ROS threaten the body's oxidative defense system, resulting in damage to biological molecules such as lipids, nucleic acids, and proteins [11].

In addition, excessive ROS produced by sperm in the asthenozoospermia group destroyed the structure and function of the mitochondria and disturbed oxidative phosphorylation, then resulted in a significant reduction in ATP synthesis. These results suggest that progressive motility of spermatozoa is closely related to sperm ATP synthesized by mitochondrial respiration [12].

MnSOD is an essential enzyme mainly presenting in the mitochondrial matrix which is called a natural scavenger and a blocker of free radical chain reaction. It plays a very important role in protecting cells from reactive oxygen-induced toxicity damage [13].

MnSOD is essential for cell survival and ATP production. Studies had demonstrated that low antioxidant enzyme activities are associated with asthenozoospermia in the dog [14]. We also found that MnSOD activity is obviously weakened in asthenozoospermic patients, which may be attributable to insufficient antioxidant enzymes to eliminate excess ROS in the body. Macanovic et al. reported that seminal plasma protein expression of superoxide dismutase (SOD) in the form of either MnSOD or CuZnSOD is proportional to sperm progressive motility [15]. This is consistent with our results.

The number of semen samples was limited, and the results of the study need to be further verified by increasing the number of samples. The viability of sperm in vitro decreases rapidly, and the oxidation index fluctuates greatly due to experimental conditions. SLIRP genetic testing could be one more way to judge male fertility. If there is obvious low gene expression or deletion, it can predict the weakening of male semen quality and the decline of fertility.

## 5. Conclusion

SLIRP regulates human male fertility. The expression of the SLIRP gene and protein in asthenospermia patients is downregulated. Oxidative damage in asthenospermia patients increases; however, the antioxidant enzyme MnSOD decreases and ATP synthesis also decreases. SLIRP and ROS generation are negatively correlated, while SLIRP and sperm progressive motility as well as energy synthesis were positively correlated. The specific pathway which SLIRP acts on human sperm requires further study.

## Figures and Tables

**Table 1:** The mean participants' age and semen parameters.

Parameter	NS (*N* = 60)	AS (*N* = 50)	*P* value
Age (y)	31.96 ± 5.22	34.50 ± 5.71	NS
Abstinence time (day)	5.56 ± 3.11	6.17 ± 3.99	NS
Volume of semen (mL)	3.87 ± 1.09	4.13 ± 1.24	NS
Concentration of spermatozoa (∗10^6^/mL)	98.73 ± 46.73	52.91 ± 25.26	<0.05
Total motility (%)	80.12	25.87	<0.05
Progressive motility (%)	68.52	17.95	<0.05
Normal morphology (%)	3.28	1.99	NS
DNA fragmentation index (%)	10.98	23.36	<0.05

NS: normospermia group; AS: asthenospermia group. ^∗^Compared to the normospermia group (^∗^*P* < 0.05).

## Data Availability

The authors declare that the data supporting the findings of this study are available within the article.
